# Quality and Safety of Marinating Breast Muscles of Hens from Organic Farming after the Laying Period with Buttermilk and Whey

**DOI:** 10.3390/ani10122393

**Published:** 2020-12-15

**Authors:** Anna Augustyńska-Prejsnar, Zofia Sokołowicz, Paweł Hanus, Małgorzata Ormian, Miroslava Kačániová

**Affiliations:** 1 Department of Animal Production and Poultry Products Evaluation, Institute of Food and Nutrition Technology, University of Rzeszow, 35-959 Rzeszow, Poland; zosokolo@ur.edu.pl (Z.S.); morm@ur.edu.pl (M.O.); 2 Department of Food Technology and Human Nutrition, Institute of Food and Nutrition Technology, University of Rzeszow, 35-959 Rzeszow, Poland; hanuspawel@gmail.com; 3 Department of Fruit Science, Viticulture and Enology, Faculty of Horticulture and Landscape Engineering, Slovak University of Agriculture, 949 76 Nitra, Slovakia; miroslava.kacaniova@gmail.com; 4 Department of Bioenergetics, Food Analysis and Microbiology, Institute of Food and Nutrition Technology, University of Rzeszow, 35-959 Rzeszow, Poland

**Keywords:** buttermilk, marinating, meat products, microbiological quality, physical traits, sensory properties, whey

## Abstract

**Simple Summary:**

Quality and safety are the most important features of poultry meat products for consumers. After the end of laying the meat of hens is hard, therefore methods of improving its tenderness are sought. Marinating is a frequently-used method used to improve the culinary qualities of a product. Synthetic additives for marinating meat are gaining increasingly less consumer acceptance. Acid whey and buttermilk are by-products of the dairy industry which are a source of many valuable components. The aim of this study was to evaluate marinating organic hen meat after the end of the laying period with buttermilk and whey for 24 and 48 h. In this article we have demonstrated that whey and buttermilk are suitable as natural marinades for marinating organic hen meat after laying period as they provide microbiological safety of the product and have a beneficial effect on tenderness and chewiness. In the sensory evaluation, they improve flavour and tenderness, which allows obtainment of a high-quality product.

**Abstract:**

The material for the study was the breast muscles of hens after the laying period which were marinated with buttermilk and acid whey for 24 and 48 h. The quality parameters of non-marinated and marinated raw and roast products were evaluated in respect of physical traits (marinade absorption, pH, colour L*a*b*, shear force, TPA texture profile analysis test), microbiological parameters and sensory characteristics. The microbiological parameters were determined as the total viable counts of mesophilic aerobic bacteria of the *Enterobacteriaceae* family and *Pseudomonas* spp. Bacterial identification was performed by MALDI-TOF MS. The study showed that marinating the breast muscles of hens after the laying period with buttermilk and whey lightened the colour (*p* < 0.05), decreased the shear force value (*p* < 0.05), and reduced hardness and chewiness (*p* < 0.05) both after 24 and 48 h of marinating compared to the control product. The 24-h time of marinating with buttermilk and whey inhibited (*p* < 0.05) the growth of aerobic bacteria and *Pseudomonas* spp. and had a positive effect on the desirable odour, the intensity and desirability of flavour as well as the roast product tenderness. Longer marinating time reduced the product palatability and decreased its microbiological safety. The obtained results suggest that the 24-h time of marinating hen meat after the laying period with buttermilk and acid whey allows to obtainment of a high-quality product.

## 1. Introduction

The basis for poultry meat production in the world is the intensive rearing of young slaughter birds (broiler chickens). Meat obtained from hens after the end of laying in intensive rearing conditions cannot compete with meat obtained from slaughter chickens, because it is meat from hens slaughtered at the age of several dozen weeks and is a by-product in the production of table eggs [1,2,3]. In recent years, an increase in the number of laying hens kept on organic farms has been observed in the EU countries [4]. Most often, hens of native breeds that are well adapted to local conditions are used for breeding under organic conditions [5]. In Poland, organic farms mostly use hens of the native breed Green-legged Partridge [3,6]. Increasing consumers’ awareness of animal welfare in various farming systems and the belief that organic products are healthier and tastier than those from conventional farming has resulted in an increased interest in organic products [7]. Due to economic and meat quality reasons, demand for organic laying hen carcasses after the laying period is high, as opposed to those from intensive farming. Meat obtained from organic farming hens after the laying period is darker in colour, has a higher nutritional value (more protein, less fat, favourable proportion of unsaturated fatty acids) as well as better taste and more intense smell compared to that from commercial farming [8,9]. Relative to the meat of broiler chickens, the meat of hens after the laying period is less tender, which may indicate the usefulness of marinating [6,10].

Marinating is a commonly-used technique in meat processing and preservation. Consumers’ expectations lead to a search for new and safe ingredients for marinades [11,12,13]. Synthetic additives for marinating meat have gained less and less consumer acceptance. Meeting the needs of consumers looking for new ingredients for marinades is the use of buttermilk and acid whey as natural marinades in the process of marinating the meat of hens after the laying period.

Buttermilk and whey were selected for marinating the breast muscles of hens after the laying period, because they are natural products that have a beneficial effect on human health. Buttermilk and acid whey are by-products of the dairy industry, resulting from the production of butter and curd cheese with the use of appropriate bacterial cultures. They are rich in proteins, lecithin, mineral salts (including calcium and phosphorus compounds), lactose, vitamins (especially B2 and A) and organic acids [14]. Acid whey contains proteins of high nutritional and biological value, the most important of which are *α-lactalbumin*, *β-lactoglobulin* (75% of all whey proteins), as well as lactoperoxidase, immunoglobulins, and lactoferrin [15]. It is characterised by high nutritional quality as well as antimicrobial and antioxidant properties [16]. Buttermilk is a low-calorie product. The high proportion of live lactic acid bacteria cultures present in these products enhances the secretion of gastric juices, stimulates digestion, and regulates digestive processes. Rzepkowska et al. [17] reports that the LAB strains isolated from organic whey have high potential for food application. By-products of the dairy industry, buttermilk and whey are now used to improve the health-promoting value of food and are of interest to the meat industry and household consumers. Moreover, they are widely available and cheap. The latest studies [15,17,18,19,20,21,22,23] show that fermented products of the dairy industry have antioxidant properties can be used in the marinating and processing of pork and beef. The lack of information in the scientific literature on the use of buttermilk and acid whey for marinating the meat of hens after the laying period prompted the authors to undertake research in this field.

The aim of this study was to assess the effect of marinating the breast muscles of organic hens after the laying period with buttermilk and acid whey and of the marinating time for improving the product quality and safety. 

## 2. Material and Methods

### 2.1. Raw Material Preparation

The material for the study were breast muscles (superficial and deep) acquired from hens of the native breed Green-legged Partridge after the first year of laying under organic farming conditions. The hens were housed and fed following regulations pertinent to organic rearing. The flock of laying hens was housed in a deep litter system, with a stocking rate of 6 birds/m^2^. The poultry house had windows and free access to grass-covered open-air of about 5 m^2^/hen. The hens were also given organic poultry feed (16.1% protein, 11,2 MJ). The birds were slaughtered in the 64th week of life. Twenty-four hours after slaughter, the breast muscles were manually trimmed from chilled carcasses (60 pieces), according to the simplified dissection by Ziołecki and Doruchowski [24]. Single breast muscles (*n* = 120) were used in the study. 

Buttermilk and acid whey came from a local manufacturer of dairy products, which were obtained directly from the production line of butter and organic cottage cheese. The dairy by-products used had a quality control certificate and were subjected to microbiological control by the manufacturer in accordance with the following standards PN-EN [25,26]. Fermented milk products were thoroughly mixed in a water bath at 40 °C, then the analysis of the chemical composition of buttermilk and whey was made using a Milk and Processes Chemical Composition Analyzer, Bentley B-150 (Bentley Systems, Exton, PA, USA). Buttermilk and whey contained, respectively: 3.93% and 0.59% protein; 4.97% and 4.65% lactose; 1.88% and 0.21% fat; 11.98% and 6.56% dry matter. The active acidity in the products was determined with a Five Easy PLUS FP20 pH meter (Mettler Toledo, Greifensee, Switzerland), the pH of the products was: Buttermilk 4.51 and whey 4.53. Total acidity was performed according to Jemaa et al. [27]. For buttermilk and whey, it was, respectively, 0.87 and 0.49 g of lactic acid/1.

Meat samples (*n* = 120) were divided into two groups: Non-marinated control group (*n* = 40) and marinated (*n* = 80). All samples were individually labelled.

### 2.2. Marinating Procedure

Two acidic marinades were prepared for marinating, containing as the main ingredient: Buttermilk (group BM) and acid whey (group W), to which sea salt (1.0%) and cane sugar were added (1.0%). The marinades were prepared 0.5 h before being used for the study and stored in refrigerated conditions (4 °C). The marinating process consisted of immersing the breast muscles of group MB (*n* = 40) and group W (*n* = 40) in the prepared marinades. The ratio between meat and marinade was fixed at 1:2. Samples were marinated for 24 h (in groups: MB *n* = 20 and W *n* = 20) and 48 h (in groups: MB *n* = 20 and W *n* = 20). The marinating process was conducted under refrigerated conditions (4 °C), in EU-certified food contact containers. Before and after the completion of the marinating process, the samples were weighed with an accuracy of 0.01 g (Ohaus V1193, Parsippany, NJ, USA). 

### 2.3. Samples Cooking 

Thermal treatment of the control (C) and marinated samples (BM and W) was based on roasting in an electric oven at 180 °C to obtain a temperature inside the sample of 78 °C ± 2 °C using a digital thermometer with an external probe. The test samples were weighed before and after the roasting process with an accuracy of 0.01 g (Ohaus V1193, Parsippany, NJ, USA).

### 2.4. Meat Quality Analyses

#### 2.4.1. Assessment of Physical Traits

Marinade absorption was shown as a percentage that was calculated from the difference of the sample weight before and after marinating. Determination of the acidity of the tested samples with a Hanna HI 99,163 pH meter consisted in inserting the electrode into the muscle and reading the value on the display. All the measurements in this study were taken by one researcher. Analysis of the colour parameters in CIE L*a*b* space was performed with a CR-400 colorimeter (Konica Minolta, Osaka, Japan) in accordance with the test methodology recommended by the device manufacturer. D65 illuminant and a standard colorimetric observer with a field of view of 2° were used for colour measurement. Colour was evaluated immediately after the samples were removed from the marinades. The tests were performed on the freshly cut cross-sectional area of the samples along the muscle fibres. Three measurements were made for each test. Meat tenderness was evaluated by shear force (Fmax) using a Zwick/Roell machine BT1-FR1 (Zwick, Breisgau, Germany), applying a Warner–Bratzler blade with a head speed of 100 mm·min-1 and a 0.2 N pre-cut force. The cutting was carried out on meat cubes with a cross section of 100 mm2 and length of 50 mm [28]. Texture profile analysis (TPA) was performed using a Texture Analyser CT3 25 (Brookfield, Middleboro, MA, USA) equipped with a cylindrical probe with a diameter of 38.1 mm and a length of 20 mm. The texture was determined in samples with dimensions of 20 mm × 20 mm × 20 mm. A test of double compression of the samples to 50% of their height was made. The speed of the roller movement during the test was 2 m/s, the gap between pressures was 2s. The TPA parameters: Hardness N (peak force during the first compression), springiness mm (speed of the test sample returning from the deformed state to the initial state), cohesiveness (strength of internal bonds forming the product framework); gumminess N (hardness × cohesiveness), and chewiness mJ (gumminess × springiness) were calculated from the force-time curves recorded for each sample using Texture Pro [29]. Weight loss (%) was calculated from the formula weight before roasting-weight after roasting/weight before roasting × 100.

#### 2.4.2. Microbiological Analysis

Breast muscles were sampled in an amount of 10 g using sterile scalpels and forceps and immediately transferred into a sterile stomacher bag, containing 90 mL of 0.1% peptone water (pH 7.0). The stomacher bag with the sample was placed in a bag mixer machine and mashed for 3 min at 20 °C. Bacteria were identified using standard microbiological methods. Anaerobic plate count (AC) was determined using Tryptocasein Soy Lab-Agar (TSA, Biocorp, Issoire, France) after incubation for 24 h at 30 °C under anaerobic conditions. The selective medium Pseudomonas Isolation Agar (PIA, Oxoid Ltd., Hampshire, UK) was used for *Pseudomonas* spp. which were incubated at 30 °C for 48 h. Bacteria of the *Enterobacteriaceae* family were counted on Violet Red Bile Glucose Agar (VRBL, Biocorp, Issoire, France), samples were incubated at 37 °C for 24 h. All plates were examined for typical colony types and morphology characteristics associated with each medium applied for incubation. All tested groups of bacteria were counted in triplicate. Adult colonies were counted after incubation, for continued identification all colonies were transferred to TSA medium which was incubated for 24 h at 37 °C. 

#### 2.4.3. Mass Spectrometry Identification of Isolates

The qualitative analysis of microbial isolates was performed with MALDI-TOF Mass Spectrometry (Bruker Daltonics, Bremen, Germany). Isolates from the agar were transferred into 300 µL of distilled water. Then, a quantity of 900 µL of ethanol was added, and the tubes with bacterial suspension in water were centrifuged for 2 min at 14,000 rpm. The supernatant was discarded, and the pellet was centrifuged repeatedly. After the remaining ethanol was removed, the pellet was allowed to dry. An amount of 10 µL of 70% formic acid was mixed with the pellet, and a 10 µL of acetonitrile was added. Tubes were centrifuged for 2 min at 14,000 rpm, and 1 µL of the supernatant was used for MALDI identification. Once dry, every spot was overlaid with 1 µL of the Cyano α4-hydroxycinnamic acid (HCCA) matrix and left to dry at room temperature before analysis. Generated spectra were analysed on a MALDI-TOF Microflex αLT (Bruker Daltonics, Bremen, Germany) instrument using Flex Control 3.4 software and Biotyper Realtime Classification 3.1 with BC-specific software. Criteria for reliable identification were a score of ≥2.0 at the species level. In the study, only bacteria whose identification result was above 2 were listed [30].

#### 2.4.4. Sensory Assessment

The evaluation of the sensory properties of the samples after thermal treatment was performed using the scaling method. The sensory analysis panel consisted of 7 people with confirmed sensory sensitivity and with at least 10 years of experience. The selection of people for the assessment team, and the training to check the sensory sensitivity of the candidates for assessors, were carried out according to the with the standards ISO [31] and [32]. The samples were assessed according to a 5-point hedonic scale according to Baryłko-Pikielna [33]. For the proper evaluation, the roast samples were cooled to room temperature and cut into slices of rectangular parallelepiped shape (1cm × 1cm × 3cm). All samples to be assessed were placed in covered vessels, marked with numerical codes. The samples were randomly assessed. Each panellist assessed a sample in three replications. Between each sample testing, the assessors took a break for 30 s, and rinsed their mouths using mineral water. The test was carried out in a properly prepared a room free from foreign odours, in appropriate temperature and lighting, in conditions enabling independent assessment, ensuring comfort for the assessors, and eliminating all distracting factors, in accordance with the applicable standards [34].

### 2.5. Statistical Analysis

Data were analysed by a two-way ANOVA using Statistica [35], to present the marinating effect (using buttermilk and whey) in 24 and 48 h. The collected data were checked for normality with the Kolmogorov–Smirnov test with Lilliefors correction. The homogeneity of variances was checked with the Brown–Forsythe test. To indicate the significance of differences between means in groups, used Tukey’s test at a 95% confidence level (α= 0.05). The results on the effect of marinating on sensory properties of roast products were verified with the use of non-parametric Kruskal–Wallis tests. Differences were considered as significant if *p* < 0.05. Table 1, Table 2, Table 3 and Table 4 show the values of arithmetic means ($\bar{x}$) and standard deviations of the examined traits (SD).

## 3. Results and Discussion

The study showed that after 48 h of marinating the marinade absorption was significantly higher than after 24 h (*p* < 0.05). Products marinated in buttermilk were characterized by higher marinade absorption (Table 1). The differences in marinade absorption may have been due to the thickness of the marinades and differences in the osmotic pressure exerted by different marinade solutions. Some scientists have noticed that sour marinades have a positive effect on water retention capacity [18]. These special properties of marinades are often associated with swelling and enhanced extractiveness of myofibrillar proteins and correlated with a decrease in pH and an increase in ionic strength [19,35,36]. Many authors [20,23,37,38] indicate that the acidity of the marinated meat depends on the pH of the marinade. In our study, the acidity of raw breast muscles marinated with buttermilk and acid whey was significantly (*p* < 0.05) lower than in the control group, which was due to the pH of the marinades. Only marinating with whey caused a decrease in the pH value after 48 h compared to marinating after 24 h (Table 1). According to Kim [23], a reduction in the pH of meat marinated with whey could result from the presence of natural buffers in the whey samples. Acidic marinating is a common method of improving the technical and functional properties of meat [39]. As expected, the pH of marinated meat products after heat treatment was lower (*p* < 0.05) than that of non-marinated products (Table 2) and corresponded to the acidity of raw marinated muscles.

Colour is one of the key quality indicators and the parameter of consumer and culinary usefulness of meat and meat products. The colour of marinated meat is related to the colour of the meat before marinating and to the pH of the marinade. The present study showed that marinating with buttermilk and whey significantly (*p* < 0.05) affected the lightening of colour of both raw products (Table 1) and those subjected to roasting (Table 2), compared to non-marinated products. The lightening of colour of marinated breast muscles may result from the greater amount of extracellular water introduced into the meat during marinating and lowering its pH. The colour change to a darker one (reduction of the L* parameter) was demonstrated after 48 h of marinating in buttermilk and in the control group. Latoch [21] showed that long marinating with buttermilk decreased L*, making pork steaks darker. Colour is one of the indicators of oxidative changes assessed in meat and meat products because it depends not only on the content of haem pigments but also on their oxidative-reduction changes. An important factor shaping the colour of meat is redox potential that determines the iron redox status placed centrally in the porphyrin ring of the myoglobin molecule. Changes in redness indicate the processes that occur during the processing and marinating of meat [19]. The dairy products used for marinating in the study, buttermilk and whey, reduced myoglobin oxidation, reducing the redness (a*) of raw meat products (Table 1). This was probably due to the protective antioxidant action of bioactive peptides in the hydrolysis of milk proteins [40]. Changes in the redox potential as a result of the addition of reducing compounds affect the transformation of myoglobin, resulting in a colour change and the release of non-haem iron from the myoglobin molecule [21]. The study by Vlahova-Vangelova [41] found no effect of marinating breast muscles of broiler chickens with whey on the colour of raw and grilled products.

Quality and health security depend on microbiological safety, where pathogenic bacteria and their toxins cannot be present in food [42]. In response to the growing demand for organic and safe meat products, methods are sought to prevent the proliferation of unfavourable microflora. In addition to traditional methods, such as freezing, an alternative may be the use of sour marinades based on products of the dairy industry containing lactic acid bacteria strains [43]. The present study showed that the use of buttermilk and acid whey as marinades in the process of marinating the breast muscles of hens after the laying period had a significant (*p* < 0.05) effect on the inhibition of an increase in the number of mesophilic aerobic bacteria and *Pseudomonas* spp. in the marinated raw product (Table 1). Lactic acid bacteria and whey proteins present in buttermilk and acid whey could reduce the growth of microorganisms [14,17,41]. Rzepkowska et al. [17] showed that organic whey contains a large number of microorganisms and a high variety of microbial groups, especially LAB ( *Lactobacillus plantarum* and *Lactobacillus fermentum* species), which exhibit antimicrobial activity against selective pathogenic bacteria. Moreover, it was proved that LAB strains possess strong activity of β-galactosidase and fermentation sugars as well as the ability to compete with other microorganisms. That the presence of lactic acid bacteria may limit the possibility of growth of saprophytic and pathogenic bacteria in a raw maturing meat product [14]. Raw poultry meat poses a microbiological hazard associated with the appearance of pathogenic bacteria. Among them, the most important are *Enterobacteriaceae* which can be considered an indicator of microbiological quality of meat [30,43,44]. In the present study, the amount of *Enterobacteriaceae* found in raw non-marinated pectoral muscles was 2.98 log cfu·g^−1^; in muscles subjected to 24-h marinating in buttermilk, the amount of these bacteria was 2.34 log cfu·g^−1^; whereas in raw muscles subjected to 24-h marinating in whey it was 2.68 log cfu·g-1 (*p* > 0.05). After 48 h of marinating, the number of *Enterobacteriaceae* was slightly increased, in non-marinated raw meat to 3.62 log cfu·g^−1^, in raw meat marinated in buttermilk to 2.96 log cfu·g^−1^ and in raw meat marinated in whey to 3.29 log cfu·g^−1^ (*p* > 0.05) (Table 1). After heat treatment was applied, the presence of *Enterobacteriaceae* was found only after 48 h in the group of non-marinated muscles (the control group), in the amount of 1.60 log cfu·g^−1^ (Table 2). The present study showed that the 48-h marinating time affected the growth of *Pseudomonas* spp. in the raw marinated product (Table 1). In the control group, an adverse effect of the marinating time on the presence of mesophilic aerobic bacteria *Enterobacteriaceae* and *Pseudomonas* spp. was noted. Microbiological tests of poultry meat after heat treatment and after 24 h of storage in refrigerated conditions did not show the presence of intestinal bacteria (Table 2). The results indicate that marinating with whey and buttermilk also inhibited *Enterobacteriaceae* and *Pseudomonas* spp., thus increasing the microbiological safety of the marinated product after heat treatment. In the study by Wójciak et al. [42] it was shown that acid whey can be used effectively to improve microbiological quality without adversely affecting organic sausage sensory quality.

**Figure 1:**
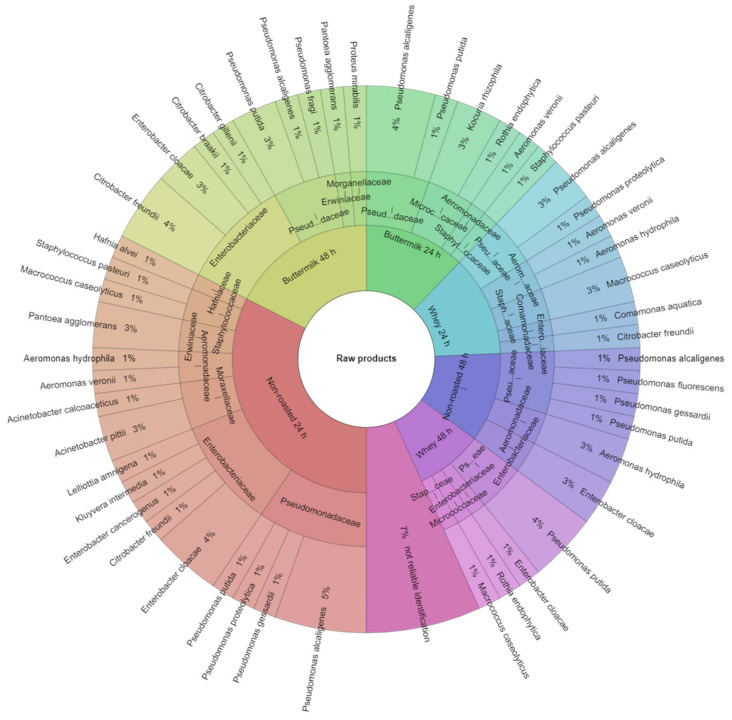
Identified species and family of bacteria in the raw products.

**Figure 2:**
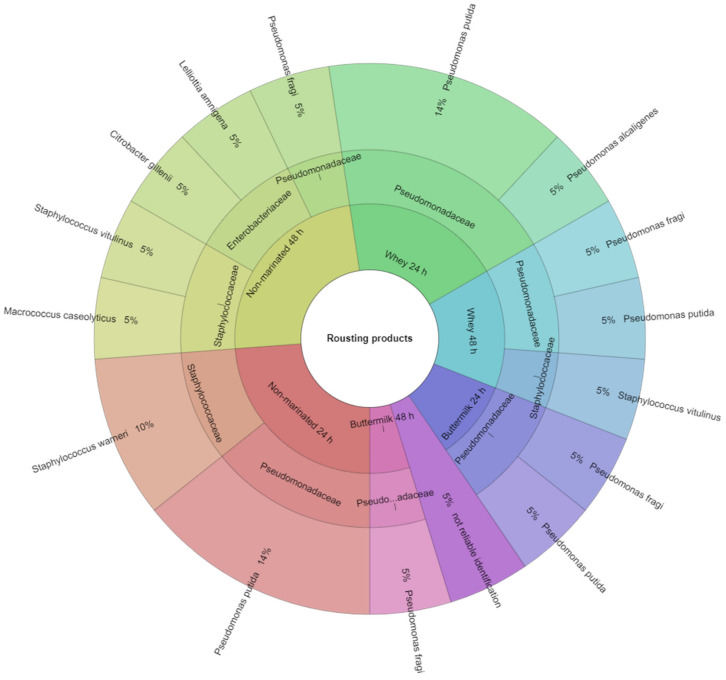
Identified species and family of bacteria isolated in the roast products.

A total of 25 species of bacteria representing 10 families were identified in the study. In this study, only bacteria whose identification result was above 2 were listed. Identification of bacteria isolated from non-marinated and marinated raw breast muscles of hens after the laying period was presented in Figure 1. 74 bacterial colonies were isolated from raw breast muscles before the marinating process, of which 93% were identified. In the *Aeromonadaceae* family, the most frequently identified bacteria were *Aeromonas hydrophila* (6%), in the *Comamonadaceae* family, the *Comamonasa aquatica* bacteria were the most frequent (1%). The *Enterobacteriaceae* family was most frequently represented by *Enterobacter cloacae* (11%). From the *Erwiniaceae* family, the most frequently identified were *Pantoea agglomerans* (4%), from the *Hafniaceae* family, *Hafnia alvei* (1%). For *Micrococcaceae,* the most frequently isolated were *Kocuria rhizophila* (3%) and *Rothia endophytica* (3%), for *Moraxellaceae*, *Acinetobacter pittii* (3%), for *Pseudomonadaceae, Pseudomonas alcaligenes* (15%). *Macrococcus caseolyticus* (6%) were the most frequently isolated species in the *Staphylococcaceae* family. The 24-h marinating in buttermilk and acid whey reduced the number of bacteria. In the raw product marinated with buttermilk, 4 families and 6 species were identified, and in the product marinated with whey, 7 species representing 6 families (Figure 1). Identification of bacteria isolated from non-marinated and marinated roast breast muscles of hens after the laying period was presented in Figure 2. Colonies isolated from roast breast muscles were identified with 95% correct identification. The *Enterobacteriaceae* family was most frequently represented by *Citobacter gillenii* (5%) and *Lelliota Amnigena* (5%), *Pseudomonadaceae* by *Pseudomonas putida* (38%), *Staphylococcus warnei* (10%), and *Staphylococcus vitulinus* (10%) were the most frequently isolated species in the *Staphylococcaceae* family. In a non-marinated product treated with the roasting process, 7 species from 3 families were identified, while in a roast product marinated with buttermilk for 24 h, only 2 species of bacteria representing 1 family were identified. After marinating with whey, the same effect was obtained. In a roast product marinated with buttermilk for 48 h, 1 species of bacteria was identified and with whey, 3 bacteria species from 2 families were identified (Figure 2). In the study, Kačániová et al. [30] and Kačániová et al. [44] showed a positive effect of the use of essential oils on inhibition of bacterial growth. Similar results were obtained in studies on poultry meat after marinating in natural marinades containing lactic acid bacteria in its own study.

Texture is one of the most important qualitative features of meat and its products. It affects the acceptance of meat among consumers. Many authors [19,21,22,23] believed that the low meat pH after marinating has positive effects on the texture. In assessing the instrumental texture of meat, the most frequently used parameter related to tenderness is the value of the maximum shear force. The present study showed that marinating the breast muscles of hens after the laying period with buttermilk and whey significantly (*p* < 0.05) decreased the value of the shear force both after 24 and 48 h of marinating. The marinating time did not affect (*p* > 0.05) the value of the shear force (Table 3). Ergezer and Gokce [12] reported a reduction in the shear value of the obtained product marinated with lactic acid. Different results of the shear force test (Warner–Bratzler) were obtained by Kim [23] using acid whey in the process of marinating beef. The compression method of texture profile analysis (TPA) mimics the conditions to which the materials are subjected throughout the mastication process [29]. The analysis of the meat texture was based on the measurement of deformations occurring during the compression of the sample, determining such parameters of the meat as: Hardness, cohesiveness, springiness, gumminess, and chewiness. The study showed that the use of buttermilk and whey had a positive effect (*p* < 0.05) on the reduction in hardness and chewiness of the roast breast muscles of hens after the laying period, compared to the muscles not subjected to the marinating process. However, no effect (*p* > 0.05) of the marinating time on the examined texture parameters was noted (Table 3). Also, in the study by Latoch et al. [19] and Latoch [21], it has been shown that the use of fermented dairy products (kefir, yogurt, and buttermilk) to marinate pork reduced the hardness and chewiness of pork loin and sous-vide steaks. Marinating the meat in acid solution causes a lowering of pH, which results in significant hydration of proteins. The tenderness increase is correlated with the increase in water retention capacity and the increased extractiveness of myofibrillar proteins. These changes can be explained by physicochemical mechanisms resulting primarily from the drop in pH and the increase in ionic strength [37,38,45]. Chewiness is described by the product of three parameters, such as: Hardness, springiness, and cohesiveness. Meat that is more tender during biting and requires less effort during chewing (it has lower hardness and chewiness parameters) may be particularly attractive to consumers [46]. The study by Żochowska and Kujawska et al. [47] showed a positive effect of marinating with kefir on the hardness and springiness of wild boar meat. The combination of acid whey and sea salt as an additive to fermented pork sausage had no effect on hardness but had a positive effect on the springiness parameter [15].

Sensory scale [pt]: For odour and flavour intensity: 5—very strong, 4—strong, 3—weak, 2—perceptible, 1—imperceptible, for odour desirability: 5—very desirable, 4—desirable, 3—neutral, 2—slightly undesirable, 1—very undesirable; for flavour desirability: 5—intense flavour of the product 4—less intense flavour, 3—little intense flavour 2—slightly perceptible foreign, sour, bitter aftertaste, 1—perceptible foreign, bitter, sour aftertaste; for product juiciness: 5—very juicy, 4—juicy, 3—slightly juicy, 2—dryish, 1—clearly dry; for tenderness: 5—very tender, 4—tender, 3—slightly tender, 2—hard, 1—very hard.

Preferable sensory properties of marinated meat products are essential for consumers. They depend on the quality of the starting product and the additives used in the marinade [12]. In the present study, it was shown that the 24-h marinating time of breast muscles of organic hens after laying, both with the use of buttermilk and whey, had a beneficial effect (*p* < 0.05) on the flavour intensity and desirability, odour desirability, and product tenderness compared to the control Products marinated with buttermilk were characterized by the highest desirability of flavour. However, the 48-h marinating time had an adverse effect on the flavour intensity of the treated products (Table 4). The study by Kumor et al. [38] proved that organic acid marinades can be used to improve the tenderness and juiciness of the meat of hens after the laying period. Kim [23] found that the use of acid whey to marinate beef improved its tenderness and juiciness compared to the control group. Also, Vlahova-Vangelova et al. [41] showed a beneficial effect of marinating with whey on the tenderness of grilled broiler chicken meat. Wójciak et al. [42] reported that whey used to marinate ripening sausage caused a higher intensity of the bitter flavour, while the other sensory characteristics did not deteriorate. The same authors [48] obtained the acidic smell of boiled sausage by using whey and mustard seeds. According to Żochowska-Kujawska et al. [47], the use of marinades containing fermented milk drinks can be considered as a method of improving the organoleptic quality of venison. The use of kefir for marinating improved the tenderness, juiciness, and overall attractiveness of wild boar meat. In the study by Wójciak et al. [22], sensory evaluation revealed that the application of acid whey or set milk as a marinade in the production of organic ripening beef enhances the feeling of good smell and sour taste.

## 4. Conclusions

The study showed the effect of the marinating time on the marinade absorption. Breast muscles marinated in buttermilk were characterized by higher absorption of the marinade after 48 h of marinating than after 24 h. Marinating with buttermilk and whey decreased the pH and lightened the colour of both raw breast muscles and those subjected to roasting, reduced the product hardness and chewiness and inhibited the growth of aerobic bacteria and *Pseudomonas* spp., increasing the microbiological safety of the marinated product compared to the control. In the sensory evaluation, a beneficial effect of marinating with buttermilk and whey on the improvement of the smell, taste and tenderness of the products was noted. The recommended marinating time is 24 h, a longer marinating period reduced the product palatability and decreased its microbiological safety. The flavour desirability was rated higher for the products marinated in buttermilk, which did not lower the acceptability of this characteristic compared to those marinated in whey. The obtained results suggest that buttermilk and acid whey can be used as marinades for the organic meat of hens after the laying period, resulting in a high-quality product.

## Figures and Tables

**Table 1:** Effect of marinating on physical and microbiological characteristics of raw products.

Parameter	Marinating Time (h)	Non-Marinated	Marinated		SEM
		Group C	Group MB	Group W	
Marinade absorption (%)	24	-	^y^ 7.72 ± 0.92	^y^6.50 ± 0.57	0.03
	48	-	^x^ 11.21 ^a^ ± 0.64	^x^ 7.20 ^b^ ± 0.48	0.04
pH	24	5.82 ^a^ ± 0.06	5.26 ^b^ ± 0.05	^y^ 5.50 ^b^ ± 0.08	0.01
	48	5.69 ^a^ ± 0.02	5.38 ^b^ ± 0.03	^x^ 5.21 ^b^ ± 0.02	0.02
Colour:					
L*, lightness	24	^x^ 56.98 ^b^ ± 2.06	^x^ 63.66 ^a^ ± 2.50	59.11 ^a^ ± 2.38	0.38
	48	^y^ 54.07 ^b^ ± 1.51	^y^ 59.45 ^a^ ± 2.08	58.31 ^a^ ± 2.57	0.40
a*, redness	24	2.17 ^a^ ± 0.52	1.29 ^b^ ± 0.36	1.72 ^b^ ± 0.36	0.07
	48	1.94 ± 0.42	1.59 ± 0.43	1.79 ± 0.65	0.06
b*, yellowness	24	5.89 ± 0.84	5.26 ± 0.87	5.24 ± 0.56	0.10
	48	6.18 ± 1.01	5.43 ± 0.95	5.81 ± 1.12	0.12
Mesophilic aerobic bacteria (log cfu·g^−1^)	24	^y^ 3.85 ^a^ ± 0.16	2.89 ^b^ ± 0.12	2.37 ^b^ ± 0.08	0.05
	48	^x^ 4.41 ^a^ ± 0.13	3.27 ^b^ ± 0.30	2.46 ^b^ ± 0.26	0.08
*Enterobacteriaceae* (log cfu·g^−1^)	24	^y^ 2.98 ± 0.14	2.34 ± 0.15	2.68 ± 0.20	0.09
	48	^x^ 3.62 ± 0.08	2.96 ± 0.10	3.29 ± 0.32	0.07
*Pseudomonas* spp. (log cfu·g^−1^)	24	^y^ 3.46 ^a^ ± 0.12	^x^ 2.46 ^b^ ± 0.21	^y^ 2.27 ^b^ ± 0.07	0.04
	48	^x^ 5.64 ^a^ ± 0.22	^x^ 3.87 ^b^ ± 0.32	^x^ 3.49 ^b^ ± 0.25	0.08

Explanations: C—control group-non-marinated; group MB—marinated in buttermilk; group W—marinated in acid whey; a, b, c—values in rows with different letters differ significantly *p* < 0.05; x, y—values in columns with different letters differ highly significantly *p* < 0.05.

**Table 2:** Effect of marinating on physical and microbiological characteristics of roast product.

Parameter	Marinating Time (h)	Non-Marinated	Marinated		SEM
		Group C	Group MB	Group W	
pH	24	5.98 ^a^ ± 0.03	5.72 ^b^ ± 0.04	5.53 ^b^ ± 0.02	0.04
	48	5.78 ^a^ ± 0.04	5.84 ^b^ ± 0.06	5.50 ^b^ ± 0.02	0.06
Weight loss (%)	24	^y^ 26.60 ± 2.56	24.51 ± 2.98	23.29 ± 3.10	0.25
	48	^x^ 35.08 ± 2.00	28.67 ± 3.40	29.53 ± 2.45	0.36
Colour:					
L*, lightness	24	77.58 ^b^ ± 1.89	81.16 ^a^ ± 2.50	80.18 ^a^ ± 1.51	0.42
	48	76.46 ± 2.50	79.65 ± 2.00	78.20 ± 2.56	0.40
a*, redness	24	2.11 ± 0.54	2.36 ± 0.62	2.01 ± 0.48	0.08
	48	1.89 ± 0.62	2.04 ± 0.43	1.84 ± 0.54	0.09
b*, yellowness	24	^y^ 10.97 ± 1.32	11.50 ± 1.54	^y^ 11.10 ± 1.60	0.26
	48	^x^ 12.36 ± 1.84	12.86 ± 2.30	^x^ 13.68 ± 2.40	0.18
Mesophilic aerobic bacteria (log cfu·g^−1^)	24	2.15 ^a^ ± 0.21	1.08 ^b^ ± 0.91	1.14 ^b^ ± 0.14	0.06
	48	2.46 ^a^ ± 0.30	1.30 ^b^ ± 0.20	1.30^b^ ± 0.18	0.04
*Enterobacteriaceae* (log cfu·g^−1^)	24 48	1.60 ± 0.08	-	-	- 0.04
*Pseudomonas* spp. (log cfu·g^−1^)	24	2.36 ^a^ ± 0.16	1.10 ^b^ ± 0.22	1.04 ^b^ ± 0.25	0.09
	48	2.42 ^a^ ± 0.35	1.33 ^b^ ± 0.25	1.47 ^b^ ± 0.14	0.08

Explanations: C—control group-non-marinated; group MB—marinated in buttermilk; group W—marinated in acid whey; a, b, c—values in rows with different letters differ significantly *p <* 0.05; x, y—values in columns with different letters differ highly significantly *p* < 0.05.

**Table 3:** Effect of marinating on texture parameters of roast products.

Parameter	Marinating Time (h)	Non-Marinated	Marinated		SEM
		Group C	Group MB	Group W	
Warner–Bratzler					
Shear force (N)	24	32.05 ^a^ ± 2.95	28.10 ^b^ ± 3.00	29.50 ^b^ ± 3.50	0.38
	48	33.25 ^a^ ± 2.70	30.70 ^b^ ± 2.60	30.65 ^b^ ± 1.98	0.40
Hardness (N)	24	25.82 ^a^ ± 2.26	17.27 ^b^ ± 3.00	19.57 ^b^ ± 2.08	0.30
	48	26.11 ^a^ ± 2.02	18.38 ^b^ ± 2.00	18.21 ^b^ ± 1.88	0.48
Cohesiveness	24	0.35 ± 0.05	0.30 ± 0.04	0.28 ± 0.08	0.02
	48	0.40 ± 0.04	0.28 ± 0.05	0.32 ± 0.05	0.03
Springiness (mm)	24	2.06 ± 0.41	2.03 ± 0.61	1.89 ± 0.52	0.06
	48	2.29 ± 3.00	2.00 ± 0.40	1.69 ± 0.50	0.05
Gumminess (N)	24	8.98 ± 0.98	5.18 ± 0.76	5.42 ± 0.76	0.03
	48	9.12 ± 0.92	5.98 ± 0.76	4.62 ± 0.90	0.04
Chewiness (mJ)	24	17.75 ^a^ ± 1.72	11.01 ^b^ ± 1.21	10.35 ^b^ ± 1.50	0.21
	48	19.56 ^a^ ± 2.30	9.20 ^b^ ± 1.80	11.80 ^b^ ± 1.80	0.28

Explanations: C—control group-non-marinated; group MB—marinated in buttermilk; group W—marinated in acid whey; a, b, c—values in rows with different letters differ significantly *p* < 0.05; x, y—values in columns with different letters differ highly significantly *p* < 0.05.

**Table 4:** Effect of marinating on sensory properties of roast products.

Parameter	Marinating Time (h)	Non-Marinated	Marinated		SEM
		Group C	Group MB	Group W	
Odour intensity	24	4.35 ± 0.48	4.60 ± 0.52	4.64 ± 0.34	0.06
	48	4.00 ± 0.38	4.38 ± 0.42	4.46 ± 0.38	0.07
Flavour intensity	24	3.88 ^b^ ± 0.40	4.78 ^a^ ± 0.44	4.60 ^a^ ± 0.46	0.08
	48	3.80 ± 0.50	4.36 ± 0.47	4.20 ± 0.40	0.06
Odour desirability	24	4.00 ^b^ ± 0.42	4.84 ^a^ ± 0.32	4.78 ^a^ ± 0.42	0.05
	48	3.90^b^ ± 0.36	4.74 ^a^ ± 0.38	4.62 ^a^ ± 0.38	0.04
Flavour desirability	24	3.80 ^c^ ± 0.42	^x^ 4.92 ^a^ ± 0.50	^x^ 4.50 ^b^ ± 0.31	0.06
	48	3.80 ± 0.38	^y^ 4.20 ± 0.40	^y^ 3.90 ± 0.37	0.07
Juiciness	24	3.96 ± 0.27	4.58 ± 0.38	4.46 ± 0.48	0.06
	48	3.90 ± 0.30	4.40 ± 0.36	4.28 ± 0.32	0.06
Tenderness	24	3.62 ^b^ ± 0.41	4.68 ^a^ ± 0.44	4.60 ^a^ ± 0.38	0.04
	48	3.80 ± 0.36	4.60 ± 0.39	4.42 ± 0.45	0.06

Explanations: C—control group – non-marinated; group MB—marinated in buttermilk; group W—marinated in acid whey; a, b, c—values in rows with different letters differ significantly *p* < 0.05; x, y—values in columns with different letters differ highly significantly *p* < 0.05.
